# Misaligned Expectations: Motivations of Medically Underserved People to Enroll in the *All of Us* Research Program

**DOI:** 10.1002/eahr.70019

**Published:** 2026-07-02

**Authors:** Carolyn P. Neuhaus, Danielle Pacia, Camila Salvagno, Johanna T. Crane

**Affiliations:** ^1^ Research scholar at The Hastings Center for Bioethics; ^2^ Student at Yale Law School; ^3^ Constituent engagement manager for Undue Medical Debt; ^4^ Professor in the Alden March Bioethics Institute at Albany Medical College

**Keywords:** human research ethics, genetic research, genetic research results, genetic information, willingness to participate, underrepresented groups, medically underserved people

## Abstract

It is important to know what motivates people, especially from groups underrepresented in biomedical research, to accept or decline participation in research studies. With this information, engagement strategies, incentives to participate, and benefits of participation can be aligned with what potential research participants value and expect from participating in research. Our project sought to identify what motivated people recruited at a Federally Qualified Health Center (FQHC) to enroll, or not, in the *All of Us* Research Program (AoU). Qualitative interviews revealed that the most common motivator was the prospect of learning information about their health, especially genetic information that might indicate inherited disease or disease risk, and opportunities for disease prevention. However, our research also revealed that the low‐income, medically underserved people typically served by FQHCs face myriad financial, social, and political barriers to reaping the potential health benefits of knowing genetic health information. There is currently a misalignment between what motivates actual and potential research participants to enroll in AoU and the ability of low‐income, medically underserved people to use genetic research results to benefit their health. Whether or not learning genetic research results leads to improved health outcomes should be approached as a question rather than an assumption. Embedding research within an unequal society remains a barrier to aligning research participants’ motivations with the benefits that participation in research can deliver.

To help ensure that engagement strategies, incentives to participate, and benefits of participation can be aligned with what potential research participants value and expect from participating in research, it is critical to understand what motivates people to accept or decline invitations to participate in biomedical research. Because existing databases from genetic research contain data primarily from people of European descent, it is especially important to understand the motivations of people from underrepresented groups who are recruited to participate in genetics research studies. However, a misalignment of motivations and expectations between potential research participants and researchers may stymie efforts to increase participation of individuals from underrepresented groups, e.g., racial and ethnic minorities, people with disabilities, and low‐income people. Inclusion of diverse groups in biomedical research is congruent with the goal of generating scientific discoveries that benefit all groups and, ideally, contribute to the redress of health inequities.^1^

Barriers to research participation among individuals from historically underrepresented groups are complex and well documented. These barriers include medical harm and bias, distrust and mistrust,^2^ fear, cultural and linguistic differences, and logistical issues like childcare, scheduling conflicts, and transportation.^3^ Unequal distribution of the downstream benefits of research across populations may give the impression that research will not fairly benefit medically underserved, underrepresented populations. Despite this potential—and perhaps likely—unequal distribution of research benefits, studies shows that many individuals from underrepresented groups are interested in participating in biomedical research. For example, although Latinos and African Americans worry about unequal burdens of research risks and access to the benefits of research findings, they also value and support inclusion of their communities in research.^4^, ^5^, ^6^ Existing qualitative studies suggest that participation in genetic research by underrepresented racial and ethnic groups may be motivated by concerns about health disparities impacting their communities, trusting relationships with researchers, concrete benefits such as monetary remuneration and health care services, and altruism.^7^, ^8^, ^9^

Our study, the Motivations Study, sought to identify factors motivating low‐income, medically underserved people to enroll, or not, in the *All of Us* Research Program (AoU). AoU is a precision medicine research project housed within the National Institutes of Health (NIH) with an aim of recruiting one million or more US‐based volunteers who reflect the country's diversity. AoU collects participants’ genomic information from their biospecimens, and environmental, physiologic, and health data from their health records, surveys, and wearable technologies. The purpose of AoU is to establish a national public resource for studying the biological, social, and environmental determinants of health and disease. Participant diversity is a founding tenet of the program, with a specific recruitment focus on underrepresented populations, especially those most affected by health disparities in the United States.^10^

The vast majority of biospecimens used in genetic research come from people of European ancestry, leading to discoveries that exclude and/or misunderstand genetic components of disease in ancestrally diverse populations.^11^, ^12^ Racial and ethnic categories are social, not biological or genetic, but they have long been used—problematically, many argue—as proxies for ancestral diversity.^13^, ^14^, ^15^, ^16^, ^17^ Because the prevalence of some DNA variants related to health, disease, and medication response varies across ancestral populations, the lack of racial diversity among those who participate in genetic research studies is thought to be a critical barrier to understanding genetic factors that may impact the health of individuals with non‐European ancestry. To address this disparity, AoU has aimed to have 75% of participants from groups underrepresented in genetic research, with half of this diversity from racial or ethnic minorities.^10^ AoU has been both criticized for at times appearing to elide the distinction between race and genetic ancestry, and praised for facilitating research that empirically demonstrates a lack of congruence between the two.^18^, ^19^

We conducted qualitative interviews with people at one federally qualified health center (FQHC) serving as an enrollment site for AoU. Like AoU, we sited

**Knowing what motivates individuals to participate in biomedical research can help researchers better understand participants’ expectations and desires regarding research participation.**

our qualitative interview project within an FQHC in order to efficiently sample people from groups underrepresented in biomedical research who are also medically underserved, and also to understand this unique research context. FQHCs and similar health centers (called “FQHC look‐alikes”) provide primary care and other health services to over 52 million people in the US; they do not typically initiate or house research studies.^20^, ^21^ These centers provide care for any individual or family, regardless of their ability to pay, with a sliding fee scale applied for under‐ and uninsured patients. Individuals and families served by these health centers are among the most financially vulnerable in the nation and often have multifaceted health and social challenges.^22^ FQHCs are typically standalone, nonprofit organizations; they are not part of hospital systems. As such, patients are referred elsewhere for diagnostic and specialist care—the types of services needed to follow up on genetic research results.

Our open‐ended qualitative interviews with people at our FQHC site provide insight into the motivations of people from underrepresented groups to enroll, or not, in AoU. Our interviewees included both enrollees in AoU and nonenrollees. AoU already tracks for internal purposes participants’ reasons for enrolling using a drop‐down menu on its electronic enrollment platform. While a drop‐down menu is a useful method for eliciting a contained response to a specific question, it cannot capture the uncertainty, ambivalence, or contradictory feelings that participation in AoU may elicit for some.^23(p.82)^ Little is known about the motivations of people who choose not to enroll in AoU. At the time of our study, AoU enrollees had the choice to opt‐in or opt‐out of receiving genetic research results if researchers analyzed their DNA sample. Participants who enrolled in 2025 or later will no longer be provided with genetic research results.^24^ Our findings and conclusion nonetheless have implications for genomics research and research ethics.

## Study Methods

We conducted one‐on‐one qualitative interviews with individuals about their motivations to participate or not participate in AoU. Enrollees and nonenrollees in AoU were included in our study. To be eligible, participants had to have prior knowledge of AoU. Those enrolled in AoU received information about the program during the informed consent process; nonenrollees were briefed on the program prior to being interviewed for our study. We began recruiting participants for our study in March 2020. We adapted our recruitment methods and process for conducting interviews during the peak years of the Covid‐19 pandemic.

Participants were recruited via in‐person table‐based recruitment (“tabling”) and word of mouth at an FQHC serving as an enrollment site for AoU. Tabling occurred in the lobby and one waiting room of the health center and was conducted by native English speakers proficient in conversational Spanish. The table displayed a poster and flyers in Spanish and English about our study, and study staff offered granola bars to those who approached. Our table was located about 20 feet from an AoU recruitment table. AoU recruitment staff referred individuals to our table; similarly, we directed individuals unfamiliar with AoU to the AoU table. Regardless of whether they chose to enroll in AoU they could return to sign up for our study, having been briefed about AoU from AoU staff to meet our eligibility criteria. In addition, we recruited Spanish speaking participants through word of mouth via the health center's promotora program, a network of Spanish‐speaking volunteers (unpaid) who educate people in the community about the health center's services. We briefed several promotoras about our study, and they spread the word. The vast majority of individuals interviewed were current patients at the FQHC; some were also promotora volunteers. Paid health center staff were excluded from our sample.

We conducted 77 interviews; we excluded from our analysis 18 transcripts in which the interviewees did not express a basic understanding of AoU. Lack of understanding was determined during the interview through questions designed to ascertain the accuracy of participants’ self‐report of having been enrolled in or briefed on AoU. We were not able to independently verify an interviewee's AoU enrollment status, due to AoU privacy policies. That is, AoU staff were not permitted to tell us whether a potential interviewee for our study was enrolled or not in AoU, or whether they had been educated about AoU by AoU study staff. This led to a high exclusion rate, because we sometimes learned during an interview that the interviewee did not meet our eligibility criteria. We excluded interviewees who reported that they were enrolled in AoU but then revealed details that were inconsistent with AoU workflows (e.g., stating that they enrolled 10 years ago, prior to the start of AoU); interviewees who were unable to distinguish between our study and AoU; and interviewees who reported they had been briefed on AoU but then were unable to describe any specifics about the study in the interview. In addition, one interviewee was excluded because she was an employee of the health center.

**Figure 1:**
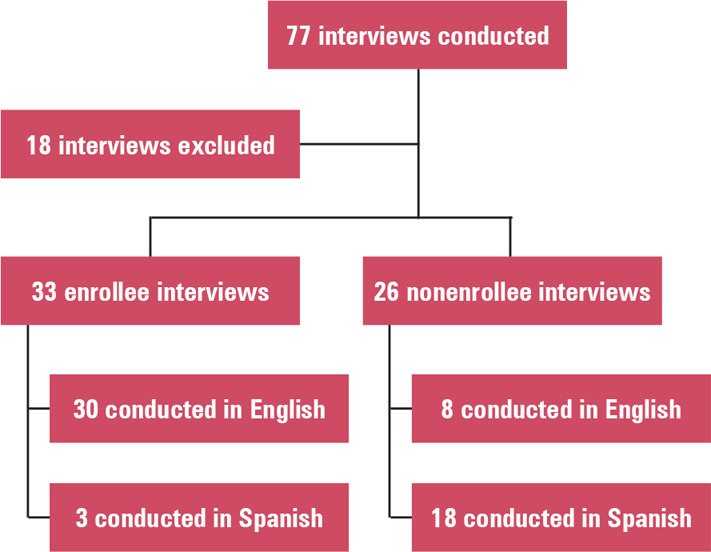
Number of Interviews by Enrollee Status and Language

Figure 1 shows the breakdown of AoU enrollees versus nonenrollees in our interview sample and the language in which the interview was conducted. Table 1 shares basic demographic information on our *included* interviews. The table documents participants’ self‐reported race and/or ethnicity, when it was provided.

**Table 1:** Self‐Reported Race or Ethnicity

	*Count*
African American	4
African Native or African Indigenous, or Black Indian	1
Black	7
Black American	1
Black and American	1
Black and American Indian	1
Black and White	1
Black or African American	1
Black, Afro‐American	1
Dominican	4
Hispanic	10
Hispanic, Columbian	1
Hispanic, Dominican Republic	1
Hispanic, Ecuadorian	1
Hispanic, Puerto Rican	1
Italian American	1
Latina or Afro‐Latina	1
Latino	1
Mixed, Columbian	1
Native American	1
Puerto Rican	1
White	5
White, Black	1
**Total self‐reported race/ethnicity**	**48**

The interview guide was developed by CPN and JTC and was informed by key informant interviews with AoU staff at the FQHC. Interviews were conducted by JTC, CPN, CS, and DP at the health center in the participant's preferred language, either English or Spanish. Spanish language interviews were conducted by a native Spanish speaker (CS). In two instances, interviews were conducted by phone; all other interviews were conducted in person. Audio recordings were professionally transcribed, and Spanish interviews were translated into English by a professional translation service. Deidentified transcripts were analyzed using an inductive, iterative approach.^25^ We developed analytic codes describing themes emerging from the data and piloted codes on a subset of transcripts to develop rules for consistent application.^26^ Transcripts were coded by a team of four researchers, all of whom were trained in qualitative analysis, using NVivo's collaboration cloud. Coding discrepancies were reconciled by consensus to ensure reliability. This study was approved by the Albany Medical College institutional review board.

The onset of the Covid‐19 pandemic at the outset of this study required considerable changes to our original methods. We originally planned to shadow AoU staff as they engaged and briefed patients in exam rooms in order to capture patients’ motivations at the time of their decision to enroll or not enroll in AoU. However, Covid restrictions limited our engagement with health center clients to tabling in the center lobby and one waiting room. As a result, we could not witness real‐time patient decision‐making about enrollment in AoU and had to rely on participant self‐report regarding their enrollment status. Some AoU participants had enrolled months or years earlier, impacting their ability to recall their motivations. In addition, levels of knowledge about AoU among our participants varied because interviewees received information about the program through multiple routes; these included engagement by AoU staff within the clinic, conversations with friends or family members, and briefing by the Motivations Study staff using publicly available AoU informational materials. These adaptations were necessary in order to conduct the study during the Covid‐19 pandemic but may limit the generalizability of our findings.

## Study Results

### Motivations to participate in AoU

Interviewees expressed a variety of motivations to participate in AoU, with many expressing more than one reason for their interest and/or enrollment in the program. The most common motivation cited among both enrollees and nonenrollees was learning health and/or ancestry information through the return of genetic research results; 44 (of 59) interviewees mentioned these forms of information as a motivator. At the time of interviewing, AoU was in the very early stages of returning genetic research results, and only two of our participants reported having genetic information available to view. One of those reported viewing ancestry information and “fun” genetic information such as cilantro distaste or aversion. The other reported an improved understanding of her family's health history; however, some of the details she shared were inconsistent with the way in which AoU typically returns genetic results, and we could not corroborate whether the results she reported viewing were provided by AoU or a different source of genetic information. Other common motivations included altruism (mentioned in 23 interviews) and the gift‐card incentive (mentioned in 19 interviews). Less common motivations included family (as beneficiaries of health or ancestry information), the desire for something to do in the community, and curiosity.

### Health information

The most common motivation by far for both enrollees and nonenrollees was learning health information about themselves. Interviewees described interest in genetic results that might indicate inherited disease or disease risk. As one interviewee explained, “If I'm pre‐disposed to breast cancer or any other things that are in my genes, it would be great to know that” (P17, enrollee, English). Family history of specific diseases, especially diabetes and cancer, were often cited as reasons for interest in genetic information; for example, one interviewee said, “I wanted to know about cancer because both my parents died of cancer” (P02, enrollee, English), and another noted that “I have a lot of family that suffer from sugar, some have died of cancer, and I want to know if one can inherit it or not” (P71, nonenrollee, Spanish).

Several interviewees also stated that knowing their own heritable disease information would be valuable for their children or other family members. “Also,” said one interviewee, “to be able to tell my kids too, about what's in their blood and what's in their DNA, whatever they do. It'll be interesting to let them know those things too, so they can be prepared when they go to the doctor and stuff” (P17, enrollee, English). Another interviewee pointed out the value of receiving heritable disease information, saying, “If it can also help part of the family because there are diseases that develop and come from the family descendants, so, if there can be a medicine that will also prevent not only for the family but also for those who come in the future, it would be important” (P75, nonenrollee, Spanish).

For some interviewees, the desire for information about disease predisposition was tied explicitly to prevention. This was true among both enrollees and nonenrollees. As one enrollee told us, “It's good to dig in, because they can tell you if you're prone to that or whatever. It's a preventive thing” (P07, enrollee, English). Some interviewees were motivated by the possibility of early treatment, with one stating, “I would like to know more about what I have inside in the case that there is medicine that can control it before it is developed” (P57, nonenrollee, Spanish). Others spoke about the opportunity to make behavior changes based on the health knowledge they might gain from the research program. “You can learn about whether you have to modify your habits, or if you have to do different things that can help you” one interviewee told us (P77, nonenrollee, Spanish). Another interviewee noted, “We're all prone to everything, but if there's anything I can avoid …. I want to be told, ‘Careful. You could have this or that condition’” (P61, nonenrollee, Spanish).

Some nonenrollees described being motivated by the possibility of getting “good news” from participation in research. As one nonenrollee explained, “I would like to hear, ‘Ma'am, you have this, and it's not bad or it's not going to affect your health’” (P62, nonenrollee, Spanish). And as another nonenrollee pointed out, the possibility of getting reassuring health information from the study was sometimes weighed against fears of being told that they were ill. They said, “Well, I would like for everything to be okay. You're always scared of what they could find because no one would like to learn that they have an illness or that they're going to have an illness. But it would also help to know that you can take better care of yourself. I think the results could help people become more aware of what we can do to fight this. And if you still don't have these conditions, you can take action to keep them from ever starting or prevent them” (P55, nonenrollee, Spanish).

A subset of respondents described AoU as a way to check and/or monitor their health, suggesting that some viewed it as akin or at least adjacent to medical care. One mentioned, “It also helps you find out about yourself, ‘cause you do get kinda like a physical” (P18, enrollee, English), while another noted that “To get the status of my medical, if it's good, bad, changing, or whatever, they'll keep track of it” (P47, nonenrollee, Spanish).

### Ancestry information

Both enrollees and nonenrollees were interested in the opportunity to receive genetic ancestry information, and for some enrollees, getting this information had been a key motivator to sign up for the program. One interviewee explained, “When they was saying what they do with the ancestors and the blood work, that's what caught my attention” (P30, enrollee, English). One interviewee expressed the point that others made about AoU participation offering the possibility of free genetic ancestry testing that would be costly in the private sector, saying, “I would love to know about my ancestry. It's very expensive to do it yourself, so I can't afford to do that” (P47, nonenrollee, English). And others, like this interviewee, expressed a general curiosity to learn more about their ethnic background, stating, “You can find out about what race you come from in what time. I'm Dominican, but I can—my nationality, my origin can be from another country” (P73, nonenrollee, Spanish). Others saw genetic ancestry information as a way to connect to heritage and ancestors. One interviewee pointed out that “We were interested especially because they were going to use the lab work to connect to our heritage. And that, to us, was very important” (P36, enrollee, English), while another said, “How can I say this? I want to learn from my ancestors…. I didn't even get to know my grandparents. Or even their grandparents from 100 years ago” (P50, nonenrollee, Spanish).

For some, the motivation to learn about ancestry was paired with the desire to learn about unknown family health history. For example, one enrollee linked racial/ethnic ancestry information to genetic health history, which she described as especially important for her and other African American people due to the history of enslavement, saying, “… see the thing that happens is we don't know, especially with Black people, I can go back as far as, in my family, to 1859. But prior to that, I don't know anything else” (P07, enrollee, English). Two enrollees who were adopted were motivated to enroll by the possibility of receiving information that would shed light on their genetic ancestry, which was unknown to them. One interviewee framed this as a desire to connect to family history by saying, “I was adopted. I wanna know where my history comes from” (P40, enrollee, English). Another sought to learn about both genetic ancestry and family genetics that might be relevant to her health, stating, “I was adopted so I don't know about my natural family. So, I'm trying to find out about my genetics. What diseases I'm pre‐disposed to and stuff like that” (P17, enrollee, English).

### Other motivations

Both enrollees and nonenrollees cited altruism as a common motivation to enroll or consider enrolling in AoU. According to one interviewee: “I feel myself helping. It's to help me and everyone else, you know what I'm saying?” (P08, enrollee, English). This motivation was often expressed as a desire to contribute to efforts to better understand and treat or prevent illnesses. An interviewee who was considering enrollment in AoU but ended up not enrolling said: “Many children have cancer. It doesn't make sense. It doesn't make sense for a child who was just born into this world to have such a serious and awful illness as that one. So, I understand that they're going to research that. And I think it's very good and important” (P50, nonenrollee, Spanish). A few interviewees who identified as Hispanic/Latino described an interest in contributing to health research that could help those who shared their ethnic background. As one interviewee explained, “If they are going to collect this information for the Latino population, a lot of times we are prone to similar diseases, so it makes sense that this will help little by little on a global level, that more information will be collected so that in the future medicine will improve” (P67, nonenrollee, Spanish). This sentiment echoed AoU's recruiting language, which at the time linked racial/ethnic diversity among participants to health research and discovery that would benefit participants’ communities.^27^, ^28^

Enrollees in AoU received a $25 gift card after contributing their biological samples to the program. Many enrollees described this incentive as a motivating factor for enrolling. However, nonenrollees rarely mentioned the gift card as a consideration about whether or not to enroll in AoU. Enrollees reported using the gift card to buy needed items, such as toilet paper or clothing for a family member and frequently described it as something they valued alongside health‐related motives. “What makes me happy is that I'm gonna get the gift card,” said one interviewee. “And God forbid if there was something wrong with me, and they did bloodwork and did a test maybe they would find something out. And who knows? Maybe they'll find something out that would help me” (P23, enrollee, English).

For a few enrollees, participating in AoU offered them something to do; an outlet or activity that they typically wouldn't have access to. One individual described being motivated out of boredom: “I'm bored at home so I don't mind” (P04, enrollee, English). Another described AoU participation as providing social interaction that was beneficial for their mental health, saying, “It's because I was very depressed, and I needed—when I couldn't talk to my therapist, I needed somebody to talk to because I'm a loner” (P37, enrollee, English). Several interviewees stated that they saw AoU as something positive for them to do with their time, because it involved engagement with the community and contribution to a collective good.

### Concerns about research participation

Interviewees also shared concerns about enrolling in AoU. The concerns emphasized by enrollees at times differed from those that were most commonly cited by nonenrollees. Because of these differences, we discuss enrollee and nonenrollee concerns separately below.

The two concerns most consistently expressed by enrollees were related to blood work and data security. Enrollees frequently noted worrying about the blood work involved with joining AoU. Sometimes this worry related to the volume of blood required by the study, as this interviewee told us: “I go to [my doctor] and only give two vials. They took six. I didn't like that at all” (P03, enrollee, English). For others, concerns over giving a blood sample had to do with fear or discomfort related to needle sticks. For some, as illustrated by comments from two interviewees, this was a general aversion to needles. They said, “I'm terrified of needles” (P04, enrollee, English); “No needles. No cutting me” (P33, enrollee, English). One individual described frustration specific to the AoU blood draw experience: “They couldn't find my vein, and they had been taking up so much time. That was my concern. I didn't like it” (P34, enrollee, English).

After blood work, the security of personal information and/or genetic data was the concern most frequently raised by enrollees. One interviewee noted: “I was more concerned about security. That was important. You have to have security” (P43, enrollee, English). As another interviewee explained, enrollment in AoU required sharing a lot of personal information with the study, raising worries about the risk of a data breach. They said, “They also had this thing where you basically gave all your information and they were putting it into the laptop and this and that. Back then is when all that craziness was going when people were stealing people's identities” (P35, enrollee, English). One enrollee described being “skittish” initially “because you don't know where this information is going” (P18, enrollee, English). Data concerns specific to one interviewee were about race and ethnicity. They said, “Because we are Native Americans, what type of protection will we have doing the DNA with them?” (P41, enrollee, English). Several enrollees who described having these concerns noted that they had had their questions satisfactorily addressed by study staff during the enrollment process. One participant said, “it worked out good cause then I realized that they were really doing what they had to do as far as security and privacy and stuff like that” (P35, enrollee, English).

Many of the nonenrollees we interviewed had learned about AoU very recently, in the days or hours preceding their interview with our study. Perhaps not surprisingly, the most common reason nonenrollees gave for not participating in AoU was that they needed more information and time to decide whether to join the program (cited in 8 interviews). One said, “Currently, I don't have enough information to be able to decide” (P76, nonenrollee, Spanish). Another noted, “I didn't know more details about what the program was specifically for, so I didn't feel confident to give more information” (P64, nonenrollee, Spanish). For this reason, we use the term “nonenrollees” rather than “decliners,” to signal that many interviewees in this category had not yet made a definitive decision about enrollment.

In addition, a nearly equal number of nonenrollees stated that they did not want or were worried about receiving health or genetic information (cited in 7 interviews). Several noted concerns about the possibility of being given distressing medical information. One interviewee specifically highlighted the potentially harmful psychological effects of getting a medically relevant genetic result, saying, “But sometimes when you learn the results, every day you're psychologically trying to figure out if you're going to get cancer or this or that” (P54, nonenrollee, Spanish). Another described weighing the risk of getting reassuring versus frightening health information, stating, “I would like to hear, ‘Ma'am, you have this, and it's not bad or it's not going to affect your health.’ Yes. But if they tell you, ‘Ma'am, you have an illness that is going to take more time or is going to start presenting itself much later in life,’ Oh, no” (P62, nonenrollee, Spanish). Others described the health and ancestry information offered by the study as something they simply did not need. Said one interviewee, “I have my primary doctor and he knows my history from way back, and stuff like that. So, I'm really not interested in the study or whatever” (P27, nonenrollee, English), and another said, “I don't need none of the family information. I know my family” (P42, nonenrollee, English).

Like enrollees, nonenrollees identified blood work (and in one case, a urine sample) as a concern. Interestingly, all the interviewees who cited the blood sample or urine sample as a reason for not enrolling (5 interviewees) were “hard” decliners. In other words, they stated they had said no to AoU recruiters and expressed certainty in their desire not to participate. Some interviewees cited anecdotes of previous negative experiences with blood draws as a reason why they were fearful. One hard decliner described a previous experience with a blood draw, saying, “She kept pricking me and pricking me …. She kept trying and trying. I said, ‘I'm gonna pass out. I'm gonna pass out.’ And, I did” (P22, nonenrollee, English). Another worried about the volume of blood required for enrollment, noting, “If you take tubes of blood out of me, I get dizzy and pass out. And, they want five tubes of blood. No. I can't do it” (P25, nonenrollee, English). One nonenrollee expressed skepticism that blood was necessary for the research program, saying, “So, as soon as they said, ‘You know, we need blood, we need from you’ … I just left…. What you need my blood for?” (P45, nonenrollee, English). These responses point to the possibility that previous negative experiences with and/or fear of needles motivates nonparticipation in research. A fear of needles is also a contributor to vaccine hesitancy.^29^ While other studies have noted that negative experiences with needles may factor into research participation decisions,^7^ this has not been studied systematically.

Small numbers of nonenrollees raised other concerns about joining AoU. A few worried about data security, though this concern was not as prominent as it was for enrollees. For example, one nonenrollee asked, “[M]aybe the purpose is good, but how are we going to be sure that that information is going to be secure?” (P67, nonenrollee, Spanish). Others cited limited time as a key concern to participating in the research program, with one explaining, “I would like to participate in the program, but I don't have much time because I have to be at work at 3:00 p.m., and we get off at 11:30 p.m. And Saturday and Sunday, I run errands and spend time with my husband, things like that” (P62, nonenrollee, Spanish). Lastly, a few nonenrollees expressed a desire to receive more than genetic results from the study, including “medical support” and “more information” regarding results and possible treatment. (AoU does provide genetic counseling to participants who receive results from the program.)

## Discussion

Knowing what motivates individuals to participate in biomedical research can help researchers better understand participants’ expectations and desires regarding research participation. Identifying areas where there is a lack of alignment between what participants (or potential participants) expect and what a research study can provide is an important step in designing studies that better address what people value and expect from research participation. This is especially important for medically underserved groups—also often underrepresented in research—who have reasons to mistrust biomedicine. For this reason, we focus our discussion mainly on areas of misalignment between our participants’ stated motivations to participate in AoU (or not) and the likelihood of those outcomes coming to fruition.

Notably, we do not see a misalignment between the desire for ancestry information and AoU's ability to meet that desire by providing ancestry information. Similarly, AoU participants motivated by the gift card, opportunities to give back to their community, and social connection also align with what AoU provides to research participants. On the flip side, the concerns raised by our interviewees, such as needle fear and time constraints, align with reasonable concerns about participation in AoU, as full enrollment does involve a blood draw and may take several hours to complete. Additionally, data breaches and re‐identification are documented risks of participating in AoU.^30^ Where our results do show misalignment is between participants’ expectations of individual and/or population health benefits from participation and the likelihood of those benefits manifesting for groups that experience myriad barriers to accessing health care.

Our results show that the prospect of receiving genetic information relevant to their health is a very common motivator for FQHC patients to consider enrolling in AoU. Citing preventing diseases such as cancers that have affected their family members, many of our interviewees expected that learning genetic information would yield improved clinical and health outcomes for themselves and genetic kin. This finding is consistent with literature about motivations to participate in genetic research and about what people value about their genetic information.^7^, ^31^ It is also consistent with NIH framings of AoU and the language used to recruit participants prior to 2025, which foregrounded learning one's genetic health information as a benefit of participating.^32^ This suggests that recent programmatic changes ending the return of genetic research results to AoU participants may have an impact on future recruitment.

AoU and similar projects have advocated for the inclusion of ancestrally diverse and historically underrepresented communities in genetic research on the premise that inclusion will offer benefits in both the short and long term. They have promoted receipt of genetic results as an *individual benefit of participation in the short term* and future medical discoveries that will reduce health disparities as a *population‐level benefit of participation in the long term*. In line with other studies of this topic, our data show that the hope for both individual level and population level benefits motivate enrollment and engagement in AoU.^7^, ^31^, ^33^ But for individual or population health benefits to be realized, individuals must be able to have access to medical care such as specialty testing and/or services not available at an FQHC. This leads us to question the assumption that genetic information translates into improved health outcomes for individuals or populations. The “consensus” position in bioethics is that research studies ought to provide participants the option to learn actionable genetic information.^34^ The fact that people want this information and believe that it will provide clinical and practical value is a strong reason to provide it, as bioethicists have argued. But does promising improved individual and population‐level outcomes as a benefit of research participation amount to misleading participants at this point in time?

To AoU's credit, at the time of our interviews, the program provided supports to assist participants in accessing potential individual‐level health benefits of research participation. AoU returned genetic research results via its online platform to participants who opted to receive them. Participants were notified via email or in‐app notification that results were available to view. AoU helped participants understand the relevance of genetic information for themselves and their families by providing free virtual genetic counseling via the telehealth company Color. For those with medically relevant research results, AoU provided free CLIA‐certified confirmation testing.

However, availing oneself of the option to view, understand, and then follow up on AoU research results takes quite a bit of participant initiative and digital literacy, not to mention reliable and affordable access to internet and internet‐connected devices: Remembering a password and logging into a web platform, coordinating and attending telehealth appointments, having a primary care provider, possibly printing out results from the AoU platform, and attending a primary care appointment with a provider who may or may not have had the opportunity to view and comprehend the meaning of the genetic research results.

Furthermore, most medically actionable research results would require specialty diagnostic services not provided within primary care, such as a colonoscopy or advanced imaging, and perhaps further specialist or preventive medical services. Elsewhere, we have described the considerable barriers that FQHC AoU participants face in accessing follow‐up care for medically relevant results, including health insurance status and cost.^35^ What most of our participants had in mind when they answered the question, “What motivated you to participate in AoU?,” was the downstream, tail end of this process: the diagnostic, medical, or pharmaceutical supports needed to prevent and manage diseases with a genetic component. That was their reported motivation. But so many financial, social, and political barriers impede its realization. Many of our AoU enrollee participants reported that they do not use the AoU app and that they struggle with email. They rely on face‐to‐face interactions with research assistants at the health center to interact with AoU, a fact corroborated by a survey of AoU staff at FQHCs.^36^ As such, the first steps of the process to reap potential individual benefits from the return of genetic results—namely, email‐based communication and app access—may already be a barrier for participants.

Beyond our concerns about barriers to learning genetic information and taking recommended actions, there is evidence that knowledge of one's genetic risk does not actually impact health behaviors such as diet, smoking, or physical activity.^37^ This raises questions such as: Does having genetic information improve health outcomes for individuals in other ways, such as through early detection and treatment? Does return of genetic results save money for individuals or the health system? Importantly, these questions of individual benefit mirror debates over the broader value of precision medicine for population health, and for medically underserved and underrepresented groups specifically.^38^ There are undeniable individual and population‐level benefits of knowing genetic information for people already exhibiting symptoms of diseases with genetic underpinnings or with known family history, including cancers or heart conditions. However, with the exception of a few conditions (namely, Huntington's disease, familial hypercholesterolemia, and highly predictive cancer genes like BRCA), the health benefits of knowing genetic information about nonsymptomatic people are unproven. There are no systematic reviews or longitudinal studies, as far as we know, that take up the question: Does receiving genetic research results improve health outcomes for research participants?

Authors of a recent qualitative interview study with precision medicine researchers raise a similar concern. Interviewees in this study, themselves precision medicine researchers across federally funded studies, questioned the benefit of returning clinically actionable results to individuals in the absence of sufficient resources for translating findings into health care for underserved populations. This prompted concern that return of results may undermine trust in precision medicine research.^39^

The main ethical problem our research uncovered is that the research and health care systems are not set up to provide the kinds of individual‐level and population‐level clinical and practical benefits that AoU participants report as having motivated them to enroll in AoU. It is nearly impossible for AoU, or any precision medicine research project, to make good on the promise of improved individual and population health management and outcomes for a large segment of the population that experiences barriers to technology, barriers to specialist care, and, additionally, are un/underinsured or simply unable to pay medical bills. FQHC patients often experience some level of food insecurity, housing insecurity, and financial strain.^40^ Following up on genetic research results may not be a top priority. The same types of issues that prevent individuals from reaping the benefits of knowing genetic information, such as enhanced monitoring, diet or exercise changes, or access to medications, may also stymie efforts to deliver population‐level benefits. There is, our research shows, a misalignment of at least some participants’ and potential participants’ motivations to enroll in AoU and the reality of what precision medicine research can deliver.

## Conclusion

There is a consensus in bioethics literature that when certain conditions are met, research studies should provide genetic results to the research participants who request them. AoU created an infrastructure to do this in a highly supportive way short of providing and/or paying for follow‐up care for AoU participants and their genetic kin. But the US health care system falls short of being able to meet enrollees’ and nonenrollees’ expectations of benefit from receiving genetic research results. This is a problem from the perspective of research ethics because it means that promises of clinical and health benefits to research participants—the same benefits that our research shows motivate their participation—may not be kept.

One interesting attempt to fix this misalignment between motivation and reality is the eMERGE study's intent to cover the medical follow‐up costs participants bear because of learning polygenic risk scores for common chronic and acute disease. However, in practice, covering these costs using research funding is not a promising or lasting strategy.^41^ On a more conceptual level, it is not the responsibility of a research project or the scientific enterprise to provide clinical care.

Our suggestion going forward is that precision medicine research projects like AoU approach the short‐term individual and long‐term population‐level health benefits of genetic research as a research *question* rather than an *assumption*. Characterizing the gap between participants’ expectations of health benefits and actual outcomes of informing nonsymptomatic people of genetic findings is a necessary first step in closing the gap.

AoU is uniquely positioned to interrogate this question by tracking the clinical outcomes of learning genetic research results. AoU tracks electronic health records (EHRs) in addition to collecting biospecimens, and so should have the necessary data. The program knows when genetic research results were made available to participants and, through participant EHRs, can in theory track next steps, including referrals and clinical outcomes. This data could provide an empirical answer to the question of whether research participation provides individual and/or population health benefits for nonsymptomatic underrepresented, underserved groups. These data should be made available to independent researchers and/or reported by AoU. The FQHCs partnering with AoU are well‐situated to track research participants’ clinical outcomes, too. With support and funding, they could launch these studies. Unfortunately, this suggestion is unlikely to be taken up in the short term due to cuts to AoU funding and scientific research funding generally in 2025. But as long as the infrastructure of the program remains in place, perhaps there is hope for the future. The aforementioned eMERGE study may produce data on the clinical outcomes of returning genetic research results in the meantime, as researchers have begun returning genetic and genomic research results to over 23,000 research participants in that study.^42^

Jabloner and Walker wrote in 2023, “efforts to diversify health research need to go hand in hand with more robust benefits for data donors (including financial benefits) and much deeper education of the genomics workforce around social dynamics of health.” They continue, “To move the needle on health equity, one cannot simply assume that more diverse datasets will automatically lead to better health care for the underserved.”^43(p.11)^ Too often, the ethics of health care are bracketed off from the ethics of research, a false division that belies the fact that it is nearly impossible to do equitable research in a context of inequitable care.^44^, ^45^ The research community, bioethicists included, should first and foremost advocate for improved access to primary, diagnostic, and specialist medical care as an imperative of *research* ethics. Personal and familial health benefits motivate people to participate in research, a reasonable expectation given the language used to incentivize participation. While additional reminders to participants about the unknown utility of learning genetic information may be warranted during the informed consent process, beyond tweaking consent processes, effort should go toward aligning the outcomes of research participation at the individual and societal levels with participants’ motivations. In the short term, this means improving tangible benefits for data donors. These may include payment to participants, offering participatory research practices for participants who desire enhanced engagement, and care navigation for people who require it based on research results (whether that's high BMI, mental health concerns, or genetic findings). Research funders should create funding streams dedicated to discovering whether and how research participants and populations enrolled in genetic research benefit from returning genetic results.

In the long term, this would mean changing the structures of science and health care so that groups who are medically underserved and underrepresented in research have reliable, affordable access to the types of services it takes for them to reap the clinical and practical benefits of knowing genetic information. This is a matter not only of who deserves health care but also what we owe to research participants—*all of us*—who contribute to scientific discovery and technological advancement.

There are a number of ethical justifications to support the notion that health care access is an imperative of research ethics. For example, social contract theory may support the notion of a right to health care and the fruits of scientific discovery because research participants have given something of themselves in the scientific endeavor. Alternatively, theories of solidarity may entail rethinking the institutions of science and health care to reduce barriers to care.^46^ Finally, an expansive notion of the learning health system that encompasses the institutions of science, technological development, and health care may support the idea that research participants are owed access to health care as a matter of research ethics. The work of fleshing out these—and other—arguments and their implications will need its own paper. For now, our results support the need for advocacy for the structures and policies that will produce health benefits for underserved people in America. Our data provide evidence that research participants from FQHCs are motivated by those health benefits: The work ahead is to outline what it takes to get there to reduce the misalignment between what motivates people to participate in genetic research and what they can reasonably expect to get out of research participation.

Community health centers that undertake and/or participate in scientific research should be engaged to figure out how to integrate their learnings into scientific discoveries and how to translate scientific discoveries into meaningful benefits for their patients. A key intermediary between the research community and populations underrepresented in research, community health centers have a wealth of knowledge about how to both improve diversity in research *and* produce positive health outcomes for marginalized and medically underserved populations.

## ACKNOWLEDGMENT

The research presented in this article was supported by the National Human Genome Research Institute and the Office of the Director of the NIH under award number R21HG010531.

We'd like to thank our participants and our FQHC partner. We owe special thanks to the AoU program staff and to the promotora program volunteers.

## DISCLOSURE

Karen Maschke, the editor‐in‐chief of *Ethics & Human Research*, was not involved in managing the peer review process for this manuscript because she was a co‐investigator on the NIH grant R21HG010531 from September 1, 2020‐August 31, 2023.
